# Telehealth Use in a Home-Based Dementia Care-Coordination Program

**DOI:** 10.1093/geroni/igaa057.633

**Published:** 2020-12-16

**Authors:** Deirdre Johnston, Melissa Reuland, Kelly Marshall, Inga Antonsdottir, Morgan Bunting, Quincy Samus

**Affiliations:** 1 Johns Hopkins Hospital, Baltimore, Maryland, United States; 2 Johns Hopkins University School of Medicine, Baltimore, Maryland, United States; 3 JHHCG, Baltimore, Maryland, United States; 4 Johns Hopkins University, Baltimore, Maryland, United States; 5 Johns Hopkins University, Halethorpe, Maryland, United States

## Abstract

In the coming decades, greater numbers of people will either have Alzheimer’s Disease or a related dementia or will take care of a family member with dementia. The dementia syndromes are associated with increased risk of medical, social, and behavioral complications in both the person with dementia (PWD) and the caregiver (CG), many of which are preventable. These complications, and the dementia itself, can impede access to care and ultimately hasten residential care placement, which can be both undesirable and costly. A nearly universal unmet need in PWD/CG dyads is dementia-specific education. Therefore, it is vital we find ways to support and provide education to CG/PWD dyads to manage dementia in the community and home setting. MIND at Home is a dementia-care model developed and tested at Johns Hopkins University School of Medicine to minimize dementia complications and delay institutionalization by training non-clinical Memory Care Coordinators (MCCs) working under clinical supervision to support and guide PWD/CG dyads in the community. MCCs collaborate with CGs and PWDs in the community using an individualized care plan structured around the dyads’ specific dementia-related needs. This presentation will describe how the MIND at Home team used handheld tablets to connect MCCs to clinicians from participants’ homes, and will report on challenges encountered, strategies to address them, and participant and caregiver satisfaction with the telehealth experience.

